# Potential problems and recommendations regarding substitution of generic antiepileptic drugs: a systematic review of literature

**DOI:** 10.1186/s40064-016-1824-2

**Published:** 2016-02-25

**Authors:** Muhammad Atif, Muhammad Azeem, Muhammad Rehan Sarwar

**Affiliations:** Department of Pharmacy, Faculty of Pharmacy and Alternative Medicine, The Islamia University of Bahawalpur, Bahawalpur, Punjab Pakistan

**Keywords:** Generic substitution, Pharmacokinetics, Bioequivalence, Bioavailability, Bioinequivalence, Narrow therapeutic index, Antiepileptic drugs

## Abstract

Despite the availability of generic antiepileptic drugs (AEDs), still patients and neurologists hesitate to make a switch due to assorted reasons. The objectives of this review were to evaluate the risks associated with the generic substitution of AEDs. In this context, we also summarized the recommendations of various international societies to treat epileptic patients. We used a number of electronic databases to identify the relevant published studies which demonstrated the potential problems and recommendations regarding generic substitution of AEDs. Of 204 articles found initially, 153 were selected for additional review. Subsequently, 68 articles were finally selected. This review concluded that potential problems linked with the generic substitution of AEDs could be bioequivalence issues, failure of drug therapy, emergence of adverse events and increase in the frequency of seizures. The reasons could be the pharmacokinetics properties of AEDs and unique characteristics of some epilepsy patients. Consequently, the generic substitution of AEDs affects the successful treatment and quality of life of the patients. Various guidelines recommend the well-controlled epileptic patients to avoid switching from brand-to-generic products, generic-to-brand products or generic to some other generic products.

## Background

Epilepsy is a familiar, chronic and critical neurologic disorder characterized by episodes (such as seizures) requiring most of the times a lifelong management (Bialer and Midha 2010; American Medical Association 2009). Being one of the most prevalent diseases, it affects about 50 million people globally and out of them 40 million are from developing countries (World Health Organization 2001). In low-income countries, its incidence may reach at a higher level of 190 in each 100,000 persons (Placencia et al. 1994). Antiepileptic drugs (AEDs) have gained much attention because of the fact that about 70 % of the epilepsy patients achieve seizure remission allowing them to live a normal life (Heaney and Sander 2007).

Trepidations about the safety and costs of the medicines have intensified the considerations to the clinical equivalence and role of the generic medicines. These are the products with same active pharmaceutical ingredient(s) (qualitatively as well as quantitatively) as that of the reference product (Van Paesschen et al. 2009). Generic medicines play an important role in patient adherence to the therapy because most of the times these are available at a considerably low price as compared to the branded products (Shrank et al. 2006; Goldman et al. 2007; Kesselheim et al. 2006). Reduction in the healthcare expenditures is crucial for economically compromised patients and those with limited health insurance facilities.

United States’ Food and Drug Administration (US FDA) states that, in 1984, about 12 % of the prescriptions included generics and this increased to 44 % in 2000. Regardless of this growth, the increment in the cost accounted for only 8 % (Bialer and Midha 2010).

Nevertheless, the major factor attributed to the extensive use of generic substituents is the reduced cost, yet low cost based generic substitution in epilepsy patients without taking into considerations the unique behavior of the disease is questionable (Jobst and Holmes 2004). Researchers have suggested that during the course of epilepsy treatment, generic substitution should either be avoided or be done with great precautions (Gidal and Tomson 2008; Krämer et al. 2007; Crawford et al. 2006) because it may lead to various complications in the patients. The reasons accountable to these problems are still not fully explored. Consequently, the American Academy of Neurology (AAN), various patient organizations and other medical associations have argued the generic substitution without the physician’s approval (Andermann et al. 2007).

The objectives of the current review were to identify potential problems arising from the generic substitution of AEDs with prime focus on their pharmacokinetics parameters, desired outcomes and recommendations.

## Review

### Search strategy and selection criteria

**Fig. 1:**
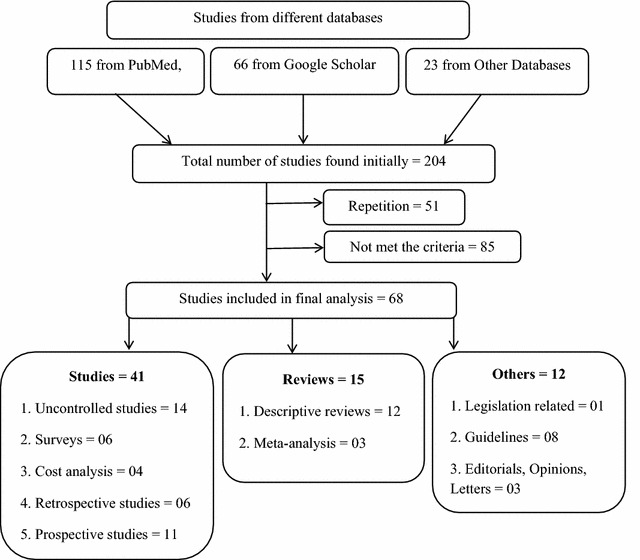
Search strategy algorithm

We explored databases (PubMed, ScienceDirect, Google Scholar, Scopus, Medline, Embase, ProQuest, SpringerLink, EconLit, etc.) from 1980 to April 2015 with these keywords: “generic substitution”, “pharmacokinetics”, “bioequivalence”, “bioavailability”, “bioinequivalence” and “narrow therapeutic index”, together with generic names of antiepileptic drugs in diverse combinations with BOOLEAN and MeSH search. Further publications were recognized by a manual search of the bibliography and reference section of related papers. Of 204 articles found initially, 153 were selected for further review. Of 153 articles, 68 were finally selected (Fig. 1).

## Results and discussion

### Potential problems with the use of generic antiepileptic drugs

It is recommended in the several guidelines to monitor the serum levels of AEDs in case generic substitution is made. This is done to confirm that the drug contact stays unaffected (Majkowski et al. 2004; Krämer et al. 2007).

If a dose adjustment is required, it should be done in such a way to avoid potential problems as a consequence of too low (therapy failure) or too high (adverse effects emergence) drug exposure. Preferably, the serum drug levels should be monitored both before and after the generic substitution of AEDs. However, practically it is not possible all the times, and may have cost implications. Furthermore, the serum drug levels of some newer AEDs are inadequately described. Nevertheless, systematically collected data of serum drug concentrations during generic substitution of AEDs offer opportunities to evaluate bioequivalence (BE) in routine care settings, and to identify the generics with potential risks to the patients.

Here, in this review we have summarized some of the problems associated with generic substituted older and newer AEDs among epileptic patients (Table 1).

**Table 1:** Potential problems reported with generic substitution of AEDs

AEDs	Potential problems	References
Carbamazepine	Increased breakthrough seizures with generic substitution	Sachdeo and Belendiuk (1987), Welty et al. (1992), Koch and Allen (1987), Hartley et al. (1991), Berg et al. (2008), Hartley et al. (1990)
	Failure of drug therapy with generic substitution	Meyer et al. (1992), Welty et al. (1992), Jain (1993)
	Toxicity and increased serum levels with generic substitution	Gilman et al. (1993), Jumao-as et al. (1989), Reunanen et al. (1992)
	Adverse effects with generics	Neuvonen (1985), Hartley et al. (1990), Olling et al. (1999), Garnett et al. (2005)
Phenytoin	Increased breakthrough seizures with generic substitution	Yamada and Welty (Yamada and Welty 2011), Berg et al. (2008)
	Toxicity and increased serum levels with generic substitution	Soryal and Richens (1992)
	Adverse effects with generics	Chen et al. (1982)
Valproate	Increased breakthrough seizures with generic substitution	Berg et al. (2008)
	Failure of drug therapy	Margolese et al. (2010), Sherr and Kelly (1998)
	Toxicity and increased serum levels with generic substitution	Levine et al. (2000)
	Adverse effects with generics	Margolese et al. (2010), Sherwood et al. (1998), Wassef et al. (2005), Zarate et al. (1999), Schwartz et al. (2000)
Leviteracetam	Increased breakthrough seizures with generic substitution	Armstrong et al. (2010), Fitzgerald and Jacobson (2011), Chaluvadi et al. (2011)
	Adverse effects with generics	(Chaluvadi et al. 2011)
Topiramate	Increased breakthrough seizures with generic substitution	Duh et al. (2009b)
	Adverse effects with generics	Pineyro-Lopez et al. (2009)
Gabapentin	Increased breakthrough seizures with generic substitution	Berg et al. (2008)
Phenobarbital	Failure of drug therapy	Bankstahl et al. (2013)
Oxcarbazepine	Increased breakthrough seizures with generic substitution	Cook et al. (2009)
Lamotrigine	Increased breakthrough seizures with generic substitution	Makus and McCormick (2007), Nielsen et al. (2008)
	Toxicity and increased serum levels with generic substitution	Srichaiya et al. (2008), Sabroe and Sabers (2008), Nielsen et al. (2008)
	Adverse effects with generics	Andermann et al. (2007), Makus and McCormick (2007)
Primidone	Increased breakthrough seizures with generic substitution	Wyllie et al. (1987)
Zonisamide	Increased breakthrough seizures with generic substitution	Berg et al. (2008)

Although, the reasons of these potential problems are still under-discussion, many researchers have proposed different hypothesis regarding the risks arising due to the generic substitution of AEDs. Three key aspects suggested by many researchers are; pharmacokinetics characteristics of AEDs, wide-ranging bioequivalence criteria and high-risk patient groups.

#### Pharmacokinetics characteristics of AEDs

The AEDs have numerous pharmacokinetics factors that may upsurge the probability of problems associated with generic substitution (Table 2) (Crawford et al. 2006; Walker and Patsalos 1995; Perucca 1999; Morselli and Franco-Morselli 1980; Bauer et al. 1982).

**Table 2:** Pharmacokinetics characteristics of AEDs which may increase the probability of problems associated with their generic substitution

AEDs	Therapeutic range	Pharmacokinetics parameters		
		Narrow therapeutic range	Low water solubility	Nonlinear pharmacokinetics
Carbamazepine	4–12 μg/ml	Yes	Yes	Yes
Phenytoin	10–20 μg/ml	Yes	Yes	Yes
Valproate	50–100 μg/ml	Yes	No	Yes
Phenobarbital	20–40 μg/ml	Yes	No	No
Ethosuximide	40–100 μg/ml	Yes	No	Yes
Gabapentin	4–20 μg/ml	Yes	No	Yes
Lamotrigine	4–20 μg/ml	No	Yes	No
Levetiracetam	5–40 μg/ml	Yes	No	No
Oxcarbazepine	10–40 μg/ml	Yes	Yes	No
Topiramate	10–20 μg/ml	Yes	Yes	No
Tiagibine	100–300 ng/ml	Yes	No	No
Vigabatrin	0.8–36 μg/ml	Yes	No	No
Primidone	5–10 μg/ml	Yes	Yes	No
Felbamate	30–100 μg/ml	Yes	Yes	No
Zonisamide	10–40 μg/ml	Yes	Yes	Yes

Due to these attributes, it is frequently asked whether it is rational to switch the AEDs and pose the patients at the risk of adverse clinical condition. For instance, compromising potential breakthrough seizures and toxicity associated with the generic substitutions of branded carbamazepine and phenytoin respectively (Gidal and Tomson 2008).

According to the FDA, a drug is categorized as NTI if the minute changes in dose or blood concentration might cause dose and blood concentration dependent severe therapeutic failures or adverse drug reactions (Yu 2011). NTI indicates that small differences in the absorption of drugs may cause or lead to substantial negative impacts on health. NTI of AEDs compels the healthcare professionals to continuously monitor the plasma levels of these drugs.

According to the prescribers, there are certain drugs that pose problems upon generic substitution, such drugs can be described as NTI (Nuwer et al. 1990). In general, the therapeutic dose of almost all AEDs vary across patients. Therefore, it is highly recommended to individualize the dose of AEDs based on the dose–response data of that particular patient (Crawford et al. 2006). This is applicable to almost all AEDs even wider therapeutic index and low toxicity profile drugs such as lamotrigine (Guberman and Corman 2000).

#### Wide ranging bioequivalence criteria

The best method to ensure therapeutic equivalency of pharmaceutical products is bioequivalence (BE). The bioequivalency of the generic products have been approved by the FDA since the enforcement of the Drug Price Competition and Patent term Restoration Act in 1984 (Hatch–Waxman Amendments) (Karki 2005). According to the FDA, when two drugs are bioequivalent, it means that both of them will provide similar and desired clinical effects. Bioequivalence can be determined by maximum concentration of a drug in the plasma (C_max_) and the area under the plasma level-time curve up to the last quantifiable concentration (AUCt) (Nightingale and Morrison 1987; Henney 1999; Bialer and Midha 2010).

The criteria set by majority of the regulatory authorities for two products to be bioequivalent is that the AUC and C_max_ ratios of both the products should fall within a range of 80–125 % with 90 % confidence intervals (CI) (Chenu et al. 2009; FDA 2003). It would be beneficial to clearly specify the size of the CI for BE studies. As for practical purposes, generics of branded drugs have AUC and Cmax ratios that are very close to 1. With significant differences in either value, it would be unlikely for the CI to lie within the range of 80–125 % (Midha and McKay 2009).

As far as two different generics of the same brand are concerned, there could be differences in their C_max_ and AUC values. Such type of deviations are very significant for the medicinal products which have NTI, poor solubility, excitatory or inhibitory effects on hepatic enzymes and/or those with non-linear pharmacokinetics (e.g. anticonvulsants) (Crawford et al. 2006; Borgheini 2003). Recently, two articles (using Monte Carlo methods) focused on the quantitative assessment of the generic AEDs, and used classic (80–125 %) and tighter (90–111.11 %) BE limits. It was verified that generic AEDs should not be considered as therapeutically equivalent products (Karalis et al. 2013, 2014).

The approval of NTI generic products based on the BE parameters is highly controversial because apparently there could be slight differences in the values but the effects could be diverse (Meredith 2003; Browne and Holmes 2001). Another important consideration in the context of generic substitution is the frequent change in the supply source of generic medicines which may compromise the condition of the patient (Meredith 2003). Change in the supply source of medicines is mainly due to availability of generic products at a lower cost. However, the complications arising from generic substitution of some medicines, for example AEDs, direct the physicians and pharmacists to select the medicines based on brand names, specifically in high risk patient groups (Table 3). The published studies have already reported that many prescribers and physicians avoided and opposed the generic substitution of the AEDs because of a greater risk of breakthrough seizures (Perucca et al. 2006; Jobst and Holmes 2004).

**Table 3:** Special categories of patients recommended for exclusion from the compulsory generic substitution (Lamy 1986; Krämer et al. 2007; Crawford et al. 2006)

Special categories	Examples
High risk patients	Extreme age groups, pregnant women, patients with multiple disorders being treated with several drugs, solitary individual, etc.
High risk diseases	Chronic diseases, diseases aggravated after the administration of drugs prescribed for co-morbid condition, etc.
High risk drugs	Narrow therapeutic index drugs, drugs requiring individualization of dose, drugs exhibiting severe drug–drug interactions, drugs with the complex therapeutic regimen, drugs initiating the prescribing cascade, etc.

#### High-risk patient groups

The problems caused by generic substitution of AEDs may particularly be significant in some specific groups of patients (Table 3). There are no systematic studies available regarding these high-risk groups, and there is little or no availability of any documented evidence that allow the quantification of the actual effect of these problems. However, physicians and pharmacists should remain alert to the problems and risks while substituting the generics. Patient-related information on their previous experiences of the generic substitution could also be beneficial to identify the risk-to-benefit ratio of generic substitution.

Examples of proposed risks to epileptic patients associated with generic substitution of medicines include; limited availability of dosage forms, drug elimination problems in renal or hepatic compromised patients, etc. AEDs have pharmacokinetics interactions with oral contraceptives so these may cause problems when used concomitantly (Crawford 2002). Generic substitution of AEDs may cause an abrupt change in the plasma concentration of the drugs, and consequently there might be failure of contraceptive therapy (Tettenborn 2006).

### Recommendations from the international societies

We have summarized the recommendations of various neurological societies in Table 4.

**Table 4:** Guidelines for generic prescription of AEDs (Krämer et al. 2007; Connock et al. 2006; Perucca et al. 2006; Network 2003; Liow et al. 2007; American Academy of Neurology 1990; Duh et al. 2009a; Bialer and Midha 2010)

Country	Organization	Principal recommendations
United States	AAN	The AAN argues the generic substitution of AEDs and advises to seek consent of attending physician
	Epilepsy Foundation	Both physician and patient should give consent and to be notified upon substitution of AEDs
	FDA	According to the FDA, a therapeutically equivalent product (either generic or brand) may be expected to have equivalent clinical effects
	American Epilepsy Society	The physicians involved in epilepsy treatment are trained for selection of appropriate AEDs and their dosages to minimize or eradicate seizures and to avoid adverse events It is done by utilizing the best available scientific evidences and clinical expertise Also, the society contradicts the formulation substitution of AEDs without obtaining approval from the physician as well as the patient
England	NICE	Be precautious while generic substitution of AEDs having complex pharmacokinetics that may cause larger differences in therapeutic effects upon minor changes in drug absorption
Germany	German chapter of ILAE	A switch must be avoided for patients having well-controlled seizures Consider a generic switch towards a lower cost AED only for the patients having poorly controlled seizures. It is better to initiate the treatment with a low-cost AED The serum drug levels should be monitored closely while switching and the patient should be informed about the potential risks
Italy	Italian chapter of ILAE	For patients exhibiting partial controlled seizures upon treatment with a brand AED, it might be appropriate to switch to a generic product The patient should be informed about the properties and nature of these products A switch is not recommended for the patients having well-controlled seizures
France	LFCE	AEDs belong to a class that may cause problems when substituted. It is recommended to avoid generic substitution of AEDs
Poland	Polish Society of Epileptology	Because of an increased risk of deterioration in epilepsy patients switching of formulations is contraindicated Pharmacists should not make substitution without informing the physicians and the physicians are responsible to make aware the patients of all the potential and possible risks
Scotland	Scottish Intercollegiate Guidelines Network	Generic substitution of AEDs should not be made as different available formulations of AEDs are not switchable
Sweden	Swedish Medicinal Products Agency	Switching between formulations may cause a poor control of seizures
Netherland	Netherlands Society of Child Neurology	The substitution of AEDs is not recommended

*AAN* American Academy of Neurology, *FDA* Food and Drug Administration, *NICE* National Institute for Health and Care Excellence, *ILAE* International League Against Epilepsy, *LFCE* Ligue Francaise Contre L’Epilepsie

## Limitations

Few AEDs for example, divalproex sodium and topiramate are also used as prophylactic agents for migraine (Chiossi et al. 2014; Steiner et al. 2007; Steiner 2005). But, due to the limited data available on the generic substitution of AEDs in migraine patients, and no such recommendations from the headache organizations (American Academy of Neurology and American Headache Society), we mainly focused on the potential problems and recommendations regarding generic substitution of AEDs in epilepsy patients.

## Conclusion and recommendations

Generic substitution is preferred to reduce the healthcare costs. However, the available literature on epilepsy indicate that substitution of AEDs is problematic, especially in certain patient groups. Generic-to-generic substitution is even not recommended based on the unavailability of BE data. Similarly, the wide-ranging criteria for bioequivalence permit variations in the drug exposure that might be clinically significant and require plasma level monitoring to avoid failure of drug therapy or incidence of adverse effects. Due to the potential risk of losing the control over seizures, various guidelines recommend that the well-controlled epileptic patients should avoid switching from brand-to-generic products, generic-to-brand products and generic-to-generic products.

As few AEDs are also used for the prophylaxis of migraine we recommend that the researchers and the associated organizations should conduct similar studies in migraine patients to evaluate the potential benefits and problems with generic substitution, and based on the results recommendations could be made for such patients.

## Abbreviations

AAN: American Academy of Neurology
AEDs: antiepileptic drugs
AUC: area under the plasma level-time curve
BE: bioequivalence
ILAE: International League Against Epilepsy
LFCE: Ligue Francaise Contre L’Epilepsie
NICE: National Institute for Health and Care Excellence
NTI: narrow therapeutic index
US FDA: United States’ Food and Drug Administration
